# Effects of temperature and humidity on the spread of COVID-19: A systematic review

**DOI:** 10.1371/journal.pone.0238339

**Published:** 2020-09-18

**Authors:** Paulo Mecenas, Renata Travassos da Rosa Moreira Bastos, Antonio Carlos Rosário Vallinoto, David Normando

**Affiliations:** 1 Department of Orthodontics, Federal University of Pará, Belém, Pará, Brazil; 2 Institute of Biological Sciences, Virology Laboratory, Federal University of Pará, Belém, Pará, Brazil; Faculty of Science, Ain Shams University (ASU), EGYPT

## Abstract

**Background:**

Faced with the global pandemic of COVID-19, declared by World Health Organization (WHO) on March 11^th^ 2020, and the need to better understand the seasonal behavior of the virus, our team conducted this systematic review to describe current knowledge about the emergence and replicability of the virus and its connection with different weather factors such as temperature and relative humidity.

**Methods:**

The review was registered with the PROSPERO database. The electronic databases PubMed, Scopus, Web of Science, Cochrane Library, LILACS, OpenGrey and Google Scholar were examined with the searches restricted to the years 2019 and 2020. Risk of bias assessment was performed using the Joanna Briggs Institute (JBI) Critical Appraisal Checklist tool. The GRADE tool was used to assess the certainty of the evidence.

**Results:**

The initial screening identified 517 articles. After examination of the full texts, seventeen studies met the review's eligibility criteria. Great homogeneity was observed in the findings regarding the effect of temperature and humidity on the seasonal viability and transmissibility of COVID-19. Cold and dry conditions were potentiating factors on the spread of the virus. After quality assessment, two studies had a high risk of bias, eleven studies were scored as moderate risk of bias, and four studies were classified as low risk of bias. The certainty of evidence was graded as low for both outcomes evaluated.

**Conclusion:**

Considering the existing scientific evidence, warm and wet climates seem to reduce the spread of COVID-19. However, these variables alone could not explain most of the variability in disease transmission. Therefore, the countries most affected by the disease should focus on health policies, even with climates less favorable to the virus. Although the certainty of the evidence generated was classified as low, there was homogeneity between the results reported by the included studies.

## Introduction

Respiratory tract infections are the most common infections worldwide, representing a source of significant morbidity and a considerable economic burden to health care [1]. The coronaviruses, *Orthocoronaviridae* sub-family, are so called for their crown-like spikes on the viral surface. They are classified into four main genus sub-groups known as *Alphacoronavius*, *Betacoronavirus*, *Gammacoronavirus*, *Deltacoronavirus*, and are able to infect human beings with an upper respiratory infections [2, 3].

A new epidemic of Severe Acute Respiratory Syndrome (SARS) Coronavirus has emerged since December 2019, namely SARS-CoV-2 or COVID-19, in Wuhan, the capital of Hubei Province, China. An outbreak of atypical pneumonia named COVID-19 caused by this virus has been reported [4, 5], and the pattern of human-to-human transmissibility of the virus has occurred nationally and internationally [6, 7].

The etiological agents have been confirmed as a new subset of coronaviruses [8]. Spread of SARS-CoV-2, like other respiratory viruses, namely its predecessor SARS-CoV, may be due to spread via droplets and contact, exposing the virus to external environmental conditions [9]. This epidemic has caused a collapse on health care services and economies of affected countries, and the overall mortality rate was estimated to be 4.7%, but in elderly patients, aged 60 or above, it can increase to up 14.8% [10]. A notable feature of SARS-CoV-2 is its predilection for transmission in the health care setting and to close family and social contacts by different manners, such as droplets, close direct or indirect contact, but the relative importance of these routes of transmission is still unclear [11]. The transmission can be affected by a number of factors, including population density, migratory flow, host immunity, medical care quality and, presumably, climate conditions (such as temperature and humidity) [12, 13].

Limited studies have investigated climate parameters as important factors that could influence the SARS-CoV-2 spread. The seasonal nature in the outbreaks of respiratory virus infections is a common phenomenon, with peaks often occurring in low temperatures, during the winter months [1]. The coronavirus can retain its infectivity up to 2 weeks in a low temperature and low humidity environment, which might facilitate the virus transmission in a community located in a subtropical climate [11]. The mechanism underlying these patterns of climate determination that lead to infection and possible disease transmission is associated with the ability of the virus to survive external environmental conditions before staying in a host [9]. Many etiological factors such as changes in host physiological susceptibility, immune system function, social behavior, and weather conditions have been suggested in this context [14].

It is supposed that high temperature and humidity, together, have a combined effect on inactivation of coronaviruses while the opposite weather condition can support prolonged survival time of the virus on surfaces and facilitate the transmission and susceptibility of the viral agent [11]. This combination may trigger an impairment of the local and systemic antiviral defense mechanisms, leading to increased host susceptibility to the respiratory viruses in winter [15].

Nevertheless, there is still divergence in the literature about the effects of temperature and humidity on the viability and transmissibility of the coronavirus infection that appeared in 2019. Faced with the global pandemic of COVID-19, declared by World Health Organization (WHO) on March 11^th^ 2020 [16], and the need to better understand the seasonal behavior of the virus, our team conducted this systematic review to describe current knowledge about the emergence and replicability of the virus and its association with different weather factors such as temperature and relative humidity. This information could be useful to develop and implement an efficient health information system with public interventions to control the incidence, and to curb the spread of COVID-19 in the world.

## Methods

### 1.1 Protocol and registration

This systematic review was registered with the PROSPERO database (CRD42020176909) and performed according with the PRISMA (Preferred Reporting Items for Systematic Reviews and Meta-Analyses) guidelines (S1 Checklist) [17].

### 1.2 Eligibility criteria

Manuscripts that evaluated the effects of different climatic conditions of temperature and/or humidity on the spread of COVID-19 were included. The search strategy was defined based on the PECOS format as follows:

Population (P): Humans diagnosed with COVID-19;
Exposition (E): Different weather conditions: humidity, temperature;
Comparison (C): Without comparison;
Outcome (O): Spread of SARS-CoV-2 (COVID-19);
Study design (S): Observational studies, prospective or retrospective, case reports, case-series.

The exclusion criteria involved studies that evaluated other upper and lower respiratory tract infections, such as Middle East Respiratory Syndrome Coronavirus (MERS-CoV), SARS-CoV and influenza. The assessment of other climatic conditions, except for temperature and humidity, was also not considered. Opinion articles, animal or laboratory studies, and literature reviews were not included.

### 1.3 Information sources

The following electronic databases were searched: PubMed, Scopus, Web of Science, Cochrane Library, LILACS, OpenGrey and Google Scholar. A hand search was also conducted by reading the references list of the included articles. No restriction on language has been applied. Date of publication has been limited to the years 2019 and 2020. The search was conducted up to March 24^th^, 2020 in all databases, and until 31^st^ March, 2020 only in Google Scholar.

### 1.4 Search strategy and study selection

The electronic searches were performed independently by two authors (P.M. and R.T.R.M.B.). In case of disagreements, a third author (D.N.) was consulted. The search strategy was developed through a combination of MeSHs, Entry Terms and Keywords related to the PECOS strategy using Boolean operators. The full search strategies for each database are illustrated in S1 File.

The citations were saved in a reference manager (EndNote, x9 version, Clarivate Analytics, Philadelphia, PA, USA). After removing duplicates, titles and abstracts were analyzed according to the eligibility criteria. The selected articles were evaluated by full text, and a final selection was conducted.

### 1.5 Data extraction

Two authors collected the data independently (P.M. and R.T.R.M.B.), extracting the following items: authors, year, location and type of study; date of COVID-19 data collection; date of meteorological data collection; sample location; weather variables; COVID-19 data sources; meteorological data sources; statistical analysis and main results. Meta-analysis was planned if there was relative homogeneity of the data and the methods for obtaining it, for each selected article.

### 1.6 Assessment of risk of bias

All the included studies were assessed for methodological rigor using the Joanna Briggs Institute (JBI) Critical Appraisal Checklist tool [18]. The checklist for cross-sectional studies uses eight criteria. The evaluation content includes: the criteria for inclusion in the sample; the study subjects and the setting described; measurement of the exposure; the objective, standard criteria used for measurement of the condition; identifying the confounding factors; the strategies to deal with confounding factors; measurement of the outcomes; and the statistical analysis used. Each component was rated as “yes”, “no”, “unclear”, or “not applicable”. With 1–3 “yes” scores, the risk of bias classification is high, 4–6 “yes” scores are moderate and 7–8 are low risk of bias. The information about the studies was extracted, synthesized from the data, and reflected in the results and conclusions of this systematic review. Two authors (P.M. and R.T.R.M.B.) independently evaluated the quality of each study, and disagreements were resolved by discussion within the review team.

### 1.7 Certainty of evidence

The included articles were given a narrative GRADE related to the outcomes assessed in this review (effects of temperature and humidity on spread of COVID-19) according to the GRADE tool (Grading of Recommendation, Assessment, Development, and Evaluation) (GRADEpro Guideline Development Tool, available online at gradepro.org) [19]. This tool considers five aspects for rating the level of evidence: design, risk of bias, consistency, directness, and precision of the studies. The level of evidence was classified as high, moderate, low, or very low. The outcomes evaluated were “Association between temperature and spread rate of COVID-19” and “Association between humidity and spread rate of COVID-19”.

## Results

### Study selection

**Fig 1:**
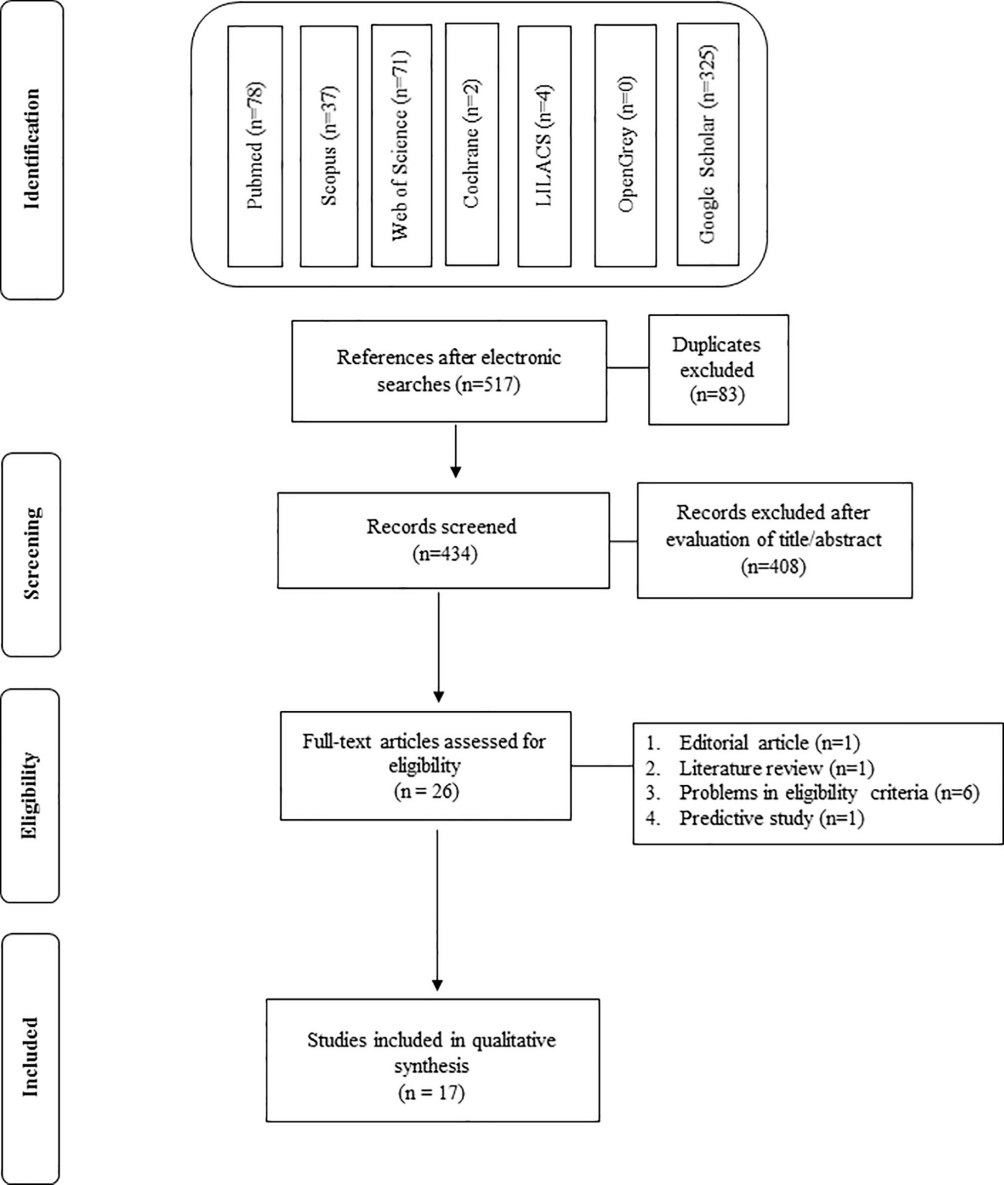
Flow diagram of study identification.

The initial searches identified 517 articles: 78 from PubMed, 37 from Scopus, 71 from Web of Science, 2 from Cochrane Library, 4 from LILACS, 0 from OpenGrey and 325 from Google Scholar. After exclusion of duplicates, 434 studies remained. After reading the titles and abstracts, 26 remaining articles were evaluated by full text and 9 were excluded. The reasons for exclusion are shown in Table 1. Seventeen studies were included and selected for qualitative analysis of risk of bias (Fig 1) [9, 20–35].

**Table 1:** List of excluded studies with reasons for exclusion.

Reference	Reason for exclusion
Boulos et al. (2020)	Editorial article.
Cai et al. (2020)	The article evaluated the mortality rate of COVID-19.
Jackson et al. (2020)	The article did not evaluate COVID-19.
Khalifa et al. (2019)	The article did not evaluate COVID-19.
Ma et al. (2020)	The article evaluated daily mortality rate of COVID-19.
Moriyama et al. (2020)	Literature review.
Neher et al. (2020)	The article did not report temperature and humidity variables.
Rai et al. (2020)	Predictive study.
Zhao et al. (2020)	The article did not evaluate COVID-19.

### Characteristics of included articles

The characteristics of the included studies are described in Table 2. All of them were retrospective observational studies that associated weather variables (temperature and humidity) with the spread of COVID-19 [9, 20–35]. Only two studies were also classified as prospective, as they both suggest future policies to prevent the spread of COVID-19 through additional control with a new vaccine [25], and the use of the best fitted predictive model of the climatic conditions of the next 12 days in 5 cities worldwide [24]. Two papers investigated the effect of temperature only on seasonal variability in transmission of COVID-19 [21, 30]. Fifteen articles evaluated the effect of the variables under study—temperature associated with humidity—in transmission of SARS-CoV-2 [9, 20, 22–29, 31–35]. It is important to highlight that two studies did not specify the type of humidity evaluated [27, 33], while 10 studies assess the relative humidity [20, 23–26, 29, 31, 32, 34, 35], four evaluated the absolute humidity [23, 26, 28, 29], one evaluated the mean humidity [22] and one evaluated the specific humidity [29]. Six studies evaluated another constant variable [20, 23–25, 27, 32], not included in this systematic review, that did not demonstrate an important factor if modeled alone in the transmission of the virus, the wind speed. It was not the objective of this systematic review to verify or discuss the statistical parameters in the manuscript. Therefore, we decided to perform a descriptive synthesis, associated with the risk of bias analysis and a narrative GRADE of evidence of the results.

**Table 2:** Summary of the data from the studies included in this review.

Authors, year, location and type of study	Date of COVID-19 data collection	Date of meteorological data collection	Sample location	Weather variables	COVID-19 data sources	Meteorological data sources	Statistical analysis	Main results
Al-Rousan et al., 2020, Turkey, retrospective observational study [20].	January 22^nd^, 2020 to February 4^th^, 2020.	January 22^nd^, 2020 to February 4^th^, 2020.	China	Temperature (Kelvin) and relative humidity (%) at two m above the ground, pressure at ground level (hPa), wind speed (m/s) and directions at 10 m above the ground, rainfall rate (kg/m^2^) snowfall rate in (kg/m^2^), snow depth (m), surface downward short-wave irradiation (watt hour/m^2^).	Johns Hopkins University Coronavirus Resource -WHO, CDC, ECDPC.	GFS Web Service-NCEP	Pearson correlation coefficient.	Weather variables showed a small effect on coronavirus transmission and no correlation can be extracted between the impact of weather and confirmed cases in all provinces. In some provinces, temperature showed a positive correlation in relation to confirmed cases and humidity demonstrated a negative correlation. In other provinces, no correlation was found.
Araújo et al., 2020, Spain/Portugal/Finland, retrospective observational study [9].	March 8^th^, 2020	January to March, 2020.	Regions with more than 5 positive cases.	Temperature (Celsius), precipitation (mm) (used as a surrogate for humidity).	Johns Hopkins University Coronavirus Resource -WHO.	CHELSA (Climatologies at high resolution for the Earth’s land surface areas)	Descriptive statistics.	The virus favors cool and dry conditions and is largely absent under extremely cold and very hot and wet conditions. This informs planning for the timing and magnitude of the likely public interventions to mitigate the adverse consequences of the coronavirus on public health.
Bannister-Tyrrell et al., 2020, Australia/France, retrospective observational study [21].	Cases reported until February 29^th^, 2020	March 4^th^, 2020.	Countries with confirmed coronavirus cases.	Temperature	Open-source line list of confirmed COVID-19.	Climate Prediction Centre (NOAA/OAR/ESRL PSD, Boulder, Colorado, USA, https://www.esrl.noaa.gov/psd/, accessed March 4^th^, 2020)	Generalized linear regression framework, ratio tests, pseudo R-squared values.	There may be seasonal variability in transmission of SARS-CoV-2, but this analysis does not imply that temperature alone is a primary driver of COVID-19 transmission. The onset of warmer weather in the northern hemisphere may modestly reduce rate of spread.
Bhattacharjee, 2020, India, retrospective observational study [32].	January 20^th^, 2020 to March 14^th^, 2020	January 20^th^, 2020 to March 14^th^, 2020	China and Italy	Maximum temperature, relative humidity, highest wind speed	WHO website and Department of Civil Protection, Italy	Local Weather Forecast, News and Conditions | Weather UndergroundOnline. (https://www.wunderground.com/. Accessed: March 9^th,^ 2020)	Pearson’s correlation coefficient.	It has been found that the relationship between the effectiveness of virus and different environmental factors is not that strong. Hence, it can be concluded that the virus shows no sign as of now, to become dormant during summer days.
Bu et al., 2020, China, retrospective observational study [22].	Not reported	October 1^st^ to December 15^th^, 2019.	China	Temperature, mean humidity.	WHO website and other public sources.	Guangdong Meteorological Observation Data Center (Wuhan 2019–2020), National Climate Center of China Meteorological Administration (China), NOAA (global temperature).	Descriptive statistics.	Warm and dry weather is favorable to the survival of the virus with a temperature range of 13–24°C, a humidity range of 50–80%, a precipitation of 30 mm/month or less. Cold air for more than a week has a significant inhibitory effect on SARS-CoV-2.
Bukhari et al., 2020, USA, retrospective observational study [23].	March 19^th^, 2020	January 20^th^, 2020 to March 19^th^, 2020.	Each country/ state (where available).	Temperature, absolute and relative humidity, wind speed.	Johns Hopkins University Coronavirus Resource Center-WHO.	‘Worldmet’ library in R from January 20^th^, 2020 to March 19^th^, 2020.	Descriptive statistics.	Based on the current data on the spread of COVID-19, the authors hypothesize that the lower number of cases in tropical countries might be due to warm humid conditions, under which the spread of the virus might be slower than has been observed for other viruses.
Chen et al., 2020, China, retrospective and prospective observational study [24].	January 20^th^, 2020 to March 11^th^, 2020.	January 20^th^, 2020 to March 11^th^, 2020.	China, Italy, Japan and other countries. USA (New York), Canada (Toronto), Italy (Milan), France (Paris), Cologne (Germany) to predict daily COVID-19 case counts in the future days.	Air temperature, relative humidity, wind speed, visibility.	WHO, CDC, ECDPC, JCDCP, DXY-COVID-19-Data.	Integrated Surface Database of USA National Centers for Environmental Information.	Loess regression interpolation, single-factor non-linear regression modeling, Pearson's correlation coefficient.	Changes in a single weather factor, such as temperature or humidity, could not correlate with the case counts very well. On the other hand, several meteorological factors combined together could describe the epidemic trend much better than single-factor models. Significant impact of daily mean temperature on the daily confirmed new case counts 14 days later. It is supposed that a sufficient time delay between exposure and confirmation is crucial for weather to exhibit its effect. There are relatively narrow temperature and humidity ranges for SARS-CoV-2 spread, there is an optimal temperature for SARS-CoV-2 at 8.07°C and most cities with high epidemic transmission of COVID-19 are located in the humidity range of 60% ~ 90%.
Gupta, 2020, India, retrospective observational study [33].	January 22^nd^, 2020 to February 16^th^, 2020	February 1^st^, 2020 to February 11^th^, 2020	China	Temperature and humidity	John Hopkins University Coronavirus Resource -WHO.	Climatic Research Unit	Fixed Effects Model Regression with Robust Standard Errors.	The results suggest that temperature has a huge effect on how rapidly the SARS-CoV-2 spreads during certain conditions. The author recommends that southern hemisphere countries prepare for increasing caseload, and northern hemisphere countries limit air conditioning.
Jiwei et al., 2020, China, retrospective and prospective observational study [25].	January 23^rd^, 2020 to February 19^th^, 2020.	Not reported	China	Air index, temperature, precipitation, relative humidity, wind power.	CDC	CMDC website	Correlation analysis, linear regression.	Higher temperature will reduce the spread of the virus. Precipitation shows low influence on COVID-19 spread. Higher relative humidity is the protection factor for the disease control.
Khattabi et al., 2020, Morocco, retrospective observational study [34].	March 17^th^, 2020	Not reported	All countries	Temperature and relative humidity	COVID-19 Coronavirus Outbreak (https://www.worldometers.info/coronavirus/)	CLIMATE-DATA (https://en.climate-data.org/)	Psychometric diagrams.	The COVID-19 has a greater impact in places where the weather is drier and colder than in places where the weather is wetter and warmer.
Luo et al., 2020, USA, retrospective observational study [26].	January 23^rd^, 2020 to February 10^th^, 2020.	January, 2020.	China, Thailand, Singapore, Japan, South Korea.	Temperature, absolute and relative humidity.	Johns Hopkins Center for Systems Science and System website-WHO, USCDCP, CDC, ECDPC, NHC, DXY-COVID-19-Data.	World Weather Online	Proxy for the reproductive number R, Clausius Clapeyron equation, Loess regression, exponential fit, linear model.	Absolute humidity and temperature are associated with local exponential growth of COVID-19 across provinces in China and other affected countries. Absolute humidity and temperature yielded a positive relationship and a slight negative relationship respectively. Changes in weather alone will not necessarily lead to declines in case counts without the implementation of extensive public health interventions.
Oliveiros et al., 2020, Portugal, retrospective observational study [27].	January 23^rd^, 2020 to March 1^st^, 2020.	January 23^rd^, 2020 to March 1^st^, 2020.	China	Temperature, humidity, precipitation, wind speed.	Not reported	Meteostat Application Programming Interface.	Descriptive statistics, exponential model, linear regression model, two way ANOVA.	The doubling time correlates positively with temperature and inversely with humidity, suggesting that a decrease in the rate of progression of COVID-19 is likely with the arrival of spring and summer in the north hemisphere. These two variables contribute to a maximum of 18% of the variation, the remaining 82% being related to other factors such as containment measures, general health policies, population density, transportation, cultural aspects.
Poirier et al., 2020, USA, retrospective observational study [28].	January 22^nd^, 2020 to February 26^th^, 2020.	January 17^th^-31^st^, 2020 and February 1^st^-15^th^, 2020.	China, Iran, Italy, Singapore, Japan, South Korea.	Near-surface air temperature, absolute humidity (near-surface water vapor density).	Johns Hopkins Center for Systems Science and Engineering website-WHO, USCDCP, CDC, ECDPC, NHC, DXY-COVID-19-Data.	ERA5 reanalysis.	Proxy for the reproductive number R, linear model with the local Rproxy, Loess regression.	Temperature showed a negative relationship, indicating that higher temperatures appeared to have lower COVID-19 transmission. Absolute humidity showed a negative relationship, indicating that locations with higher absolute humidity experienced lower transmission. Changes in weather alone will not necessarily lead to declines in case count without the implementation of extensive public health interventions.
Sajadi et al., 2020, USA/Iran, retrospective observational study [29].	Not reported	Reanalysis data for 2019 and January-February 2020.	Country-wide (epicenters of disease): South Korea, Japan, Iran, Italy, USA, Spain, France.	Two-meter temperatures, relative humidity, specific humidity, absolute humidity.	Johns Hopkins Center for Systems Science and Engineering.	ERA5 reanalysis.	Mann-Whitney and linear regression.	The combined profile of having low average temperatures and specific humidity tightly clusters all the cities with significant outbreaks as of March 10^th^, 2020 compared to other cities without COVID-19 cases. The distribution of significant community outbreaks along restricted latitude, temperature, and humidity are consistent with the behavior of a seasonal respiratory virus. Using weather modeling, it may be possible to predict the regions most likely to be at higher risk of significant community spread of COVID-19 in the upcoming weeks, allowing for concentration of public health efforts on surveillance and containment.
Shi et al., 2020, retrospective observational study [35].	January 20^th^, 2020 and February 29^th^, 2020.	January 20^th^, 2020 and February 29^th^, 2020.	Thirty one provincial-level regions in mainland China and Wuhan city.	Daily temperatures and relative humidity	CNHC using the CoV2019 package (http://www.nhc.gov.cn/)	Meteorological authority in mainland China.	Clausius-Clapeyron relation equation, incidence and the common logarithm of numbers, weighted regression and smoothing scatterplot (LOESS), distributed lag nonlinear models (DLNMs), M-SEIR model.	Lower and higher temperatures might be positive to decrease the COVID-19 incidence. The COVID-19 outbreak would not last for a long period of time with the increase of temperature, but the scale of the outbreak would be influenced by the measures taken among countries.
Wang et al., 2020, China, retrospective observational study (time-space cross-sectional study) [30].	January 20^th^, 2020 to February 4^th^, 2020.	January 1^st^, 2020 to January 30^th^, 2020.	All cities and regions affected by COVID-19 in the world (China and 26 overseas countries).	Temperature	Official websites of the Health Commissions at all levels in China and the health authorities of overseas countries.	Meteorological authority in China and in other countries.	Descriptive statistics, Log-transformation, restricted cubic spline function, generalized linear mixture model.	Temperature has significant impact on the transmission of COVID-19. There might be a nonlinear dose-response relationship between the two, indicating that there is a best temperature contributing to its transmission and that low temperature is beneficial to the viral transmission. For countries and regions with a lower temperature, strict prevention and control measures should be continued to prevent future reversal of the epidemic.
Wang et al., 2020, China, retrospective observational study [31].	Before January 24^th^, 2020.	January 21^st^, 2020 to January 23^rd^, 2020.	China	Temperature, relative humidity.	CDC	699 meteorological stations in China (if a city does not have a meteorological station inside it, the closest station is used instead).	Weibull distribution using the Maximum Likelihood Estimation (MLE) method, daily effective reproductive number R, Ordinary Least Square (OLS) method.	High temperature and high relative humidity significantly reduce the transmission of COVID-19, respectively, even after controlling for population density and GDP per capita of cities. It indicates that the arrival of summer and rainy season in the northern hemisphere can effectively reduce the transmission of the COVID-19.

WHO–World Health Organization; CDC–Chinese Center for Disease Control and Prevention; ECDPC–European Centre for Disease Prevention and Control; GFS–Global Forecast System; NCEP–National Centers for Environmental Prediction; NOAA–USA National Center for Environmental Forecasting; USA—United States of America; JCDCP–Japan Center for Disease Control and Prevention; DXY-COVID-19-Data–Chinese website that aggregates national and local CDC situation reports; CMCD–China Meteorological Data Service Center; USCDCP–U.S. Centers for Disease Control and Prevention; NHC–Chinese National Health Center; ERA5 reanalysis—a state-of-the-art data product produced at the European Centre for Medium-Range Weather Forecasts.

Great heterogeneity was observed in relation to the displayed variables, that included other weather conditions beyond temperature and humidity [20, 23–25, 27, 32] like wind speed [20, 23–25, 27, 32], visibility [24], precipitation [25, 27], pressure [20], rainfall rate [20], snowfall rate [20], snow depth [20], surface downward short-wave irradiation [20]. A heterogeneity was also observed regarding the date of data collection, both in relation to the location studied, and the date of epidemiological data collection of COVID-19 and climatic conditions.

### Results of individual studies

Great homogeneity was observed in the results of the effect of temperature and humidity in the seasonal variability and spread of the virus. Sixteen articles selected for final analysis were unanimous in stating that cool and dry conditions were potentiating factors for the spread of COVID-19 [9, 20–31, 33–35], with the spread being largely absent under extremely cold and very hot and wet conditions. Only one article reported no strong effect of temperature and humidity in the spread of the virus, nevertheless, the statistical analysis of this study was inadequate, compromising its conclusion [32].

It was also noticed that several meteorological factors combined could better describe the epidemic trend than when a single variable was analyzed [21, 24]. In addition, confounding factors as public health policies on surveillance and containment, social isolation campaigns (home quarantine strategy), including with patients’ families, socio-economic development contributes to controlling the spread of the virus around the world [9, 20, 23–29, 31, 33, 35]. Moreover, controlling population density (less crowded cities) [31] and movement [24], travel limitations [26, 28], increasing the number of medical staff and hospitals, isolating all the suspected cases, understanding the method of each patient's infection, combining the medical history of the patients with current diagnosis to extract information about the virus [20], are important measures to combat the new coronavirus.

It was verified, in countries with virus transmission under control like Korea, that the widespread testing to identify potential COVID-19 positive subjects, including asymptomatic ones, could reduce transmission [23].

Regarding the review process, sixteen of the selected articles have not yet gone through the peer review process. This must be taken into consideration when making any inference from their conclusion [9, 20–24, 26–35].

After considering all these factors, we can infer that confounding variables (like population density, purchasing power [31], public health interventions [28], containment measures, general health policies, population density, transportation, cultural aspects [27]) play an important and significant role in the spread of COVID-19.

### Synthesis of results

A meta-analysis was not performed due to the heterogeneity of the methods, locations, and information provided in the related articles investigating the proposed objectives. Additionally, differing units of measure, variables and statistical methods did not allow meaningful comparisons. Only simple and descriptive comparisons were reported, beyond the risk of bias and narrative GRADE of evidence of the results.

### Risk of bias assessment

The risk of bias in two included studies was classified as high [22, 32], eleven articles showed a moderate risk of bias [9, 20, 21, 23–27, 29, 30, 34], and four showed a low risk of bias (Table 3) [28, 31, 33, 35]. The questions that received more "no" answers, indicating study limitations, were: “Were confounding factors identified?” [9, 20–24, 26, 29, 30, 32, 34, 35]; “Were strategies to deal with confounding factors stated?” [9, 20–26, 29, 30, 32, 34, 35]; “Was appropriate statistical analysis used?” [9, 20, 22, 23, 25, 29, 32]. Other less frequent problems were observed in the questions: “Were the criteria for inclusion in the sample clearly defined?” [21]; “Was the exposure measured in a valid and reliable way?” [22, 27]; “Were objective, standard criteria used for measurement of the condition?” [20, 27, 32]; “Were the outcomes measured in a valid and reliable way?” [9, 22, 27, 32].

**Table 3:** Risk of bias assessment of the studies included in the review.

Questions/Author	Al-Rousan, 2020 [20]	Araújo, 2020 [9]	Bannister-Tyrrell, 2020 [21]	Bhatta-charjee, 2020 [32]	Bu, 2020 [22]	Bukhari, 2020 [23]	Chen, 2020 [24]	Gupta, 2020 [33]	Jiwei, 2020 [25]	Khattabi, 2020 [34]	Luo, 2020 [26]	Oliveiros, 2020 [27]	Poirier, 2020 [28]	Sajadi, 2020 [29]	Shi, 2020 [35]	Wang, 2020 [30]	Wang, 2020 [31]
1. Were the criteria for inclusion in the sample clearly defined?	Yes	Yes	No	Yes	Yes	Yes	Yes	Yes	Yes	Yes	Yes	Yes	Yes	Yes	Yes	Yes	Yes
2. Were the study subjects and the setting described in detail?	Yes	Yes	Yes	Yes	Yes	Yes	Yes	Yes	Yes	Yes	Yes	Yes	Yes	Yes	Yes	Yes	Yes
3. Was the exposure measured in a valid and reliable way?	Yes	Yes	Yes	Yes	No	Yes	Yes	Yes	Yes	Yes	Yes	No	Yes	Yes	Yes	Yes	Yes
4. Were objective, standard criteria used for measurement of the condition?	No	Yes	Yes	No	Yes	Yes	Yes	Yes	Yes	Yes	Yes	No	Yes	Yes	Yes	Yes	Yes
5. Were confounding factors identified?	No	No	No	No	No	No	No	Yes	Yes	No	No	Yes	Yes	No	No	No	Yes
6. Were strategies to deal with confounding factors stated?	No	No	Yes	No	No	No	No	Yes	No	No	No	Yes	Yes	No	Yes	No	Yes
7. Were the outcomes measured in a valid and reliable way?	Yes	No	Yes	No	No	Yes	Yes	Yes	Yes	Yes	Yes	No	Yes	Yes	Yes	Yes	Yes
8. Was appropriate statistical analysis used?	No	No	Yes	No	No	No	Yes	Yes	No	Yes	Yes	Yes	Yes	No	Yes	Yes	Yes
Risk of Bias	Mod.	Mod.	Mod.	High	High.	Mod.	Mod.	Low	Mod.	Mod.	Mod.	Mod.	Low	Mod.	Low	Mod.	Low

### Level of evidence

The evaluation of the certainty of the evidence according to GRADE is described in Table 4. The level of certainty of outcomes evaluated in this systematic review–“Association between temperature and spread rate of COVID-19” and “Association between humidity and spread rate of COVID-19”–were classified as “low”. Since the studies are observational and presented a considerable risk of bias in some criteria assessed, the certainty of the evidence several studies did not consider variables such as migration patterns and isolation policies in their results generated received this classification. Moreover, one study did not show a significant effect of the variables under study on the spread of the new coronavirus [32].

**Table 4:** Narrative GRADE evidence profile table.

Outcomes	Impact	Nº of Studies	Certainty of the evidence (GRADE)
Association between temperature and spread rate of COVID-19	Of the seventeen articles evaluated, sixteen showed some effect of temperature on the transmission rate. Except for one, which concluded that temperature has no effect on SARS-CoV-2 transmission, the other sixteen found that warmer climates are less likely to spread the virus. Studies with more robust statistical analysis, which used multivariate tests, showed that variables such as migration patterns, public isolation policies, population density, and cultural aspects, the temperature seems to have less impact.	(17 OBSERVATIONAL STUDIES)	⨁⨁◯◯ LOW ^a^
Association between humidity and spread rate of COVID-19	Fourteen manuscripts that investigated the effect of humidity on the transmission of SARS-CoV-2 demonstrated an association between variables. Only one article reported no effect of humidity on the spread of the virus, while the other fourteen showed that wetter climates inhibit the virus spread. As with the temperature, studies with more robust statistical analysis, which used multivariate tests, showed that the adjustment for confounding factors decreases the impact of humidity on the transmission of COVID-19.	(15 OBSERVATIONAL STUDIES)	⨁⨁◯◯ LOW ^a^

a. Several studies did not consider variables such as migration patterns and isolation policies in their results, factors that directly impact on the spread rate of SARS-CoV-2/COVID-19.

## Discussion

The results of the articles included in this systematic review indicate that the spread of COVID-19 may be influenced by climatic variables such as temperature and humidity. Apparently, warmer and humid climates may show less transmission of the SARS-CoV-2 virus. Although the certainty of evidence generated was low, due to the observational design of the studies and the inherent risk of bias, overall great homogeneity of the results was observed among the included studies.

Furthermore, the spread of types of diseases caused by betacoronavirus, such as SARS-CoV-1 [11] and MERS-CoV [36], have already been shown to suffer the impact of climatic conditions. In both these coronoviruses, hot and humid climates demonstrated the ability to decrease the viability of these viruses, while in places with low temperature and humidity there was greater viral stability. The favorable cold and dry weather conditions facilitates the spread of the coronaviruses and seems to be the same for SARS-CoV-2 virus, as it was observed homogeneously in the included papers.

### Summary of evidence

Seventeen articles were included in this systematic review, and methodological issues were identified [9, 20–35]. Regarding the classification of the articles and their score evaluated by the JBI Critical Appraisal Checklist tool [18], two studies were classified as high risk of bias [22, 32], eleven articles showed a moderate risk of bias [9, 20, 21, 23–27, 29, 30, 34], and four a low risk of bias [28, 31, 33, 35]. This was due, especially, to limitations in the “identification of the confounding factors” that could interfere with the final analysis of the included articles. These many confounder weather variables, associated with public health policy, are the major limitations of this systematic review, as a simple correlation statistic design cannot be done in order to establish a direct cause-effect relationship of temperature and humidity in the spread of the new coronavirus, which implied in the negative classification of the item “the statistical analysis used”. The items “the criteria for inclusion in the sample” and “the study subjects and the setting described”, both related to sample selection criteria, received a positive rating in almost all selected articles. A reasonable explanation for this fact would be that due to the urgency of the world situation with the rapid spread of COVID-19 worldwide and the need to search for immediate responses to contain the pandemic, secondary well-founded data available was used at the moment that the studies were realized, both in relation to the epidemiology of the new coronavirus, and to the climatic conditions, in an attempt to verify a possible association between them. Finally, even considering that the search period on electronical databases were performed before the outbreak in some countries, the results can be extrapolated for the spread characteristics of COVID-19 around the world. A possible search bias did not influence negatively the results, that remained consistent and homogeneous with the most recent data [37].

The certainty of evidence of clinical outcomes was also graded using the GRADE tool [19]. The evidence was scored as low because of the study designs, classified as observational cross-sectional studies [9, 20–35], due to the effect of other confounding variables on temperature and humidity in the spread of the virus, which are not identified in some studies [9, 20–24, 26, 29, 30, 32, 34, 35], and because one selected article did not find a positive strong association between the spread of the new coronavirus with temperature and humidity [32]. One of the main limitations of this study was the use of a simple correlation in its statistical analysis, which does not allow the establishment of a cause and effect relationship between the studied variables [32]. However, due to homogeneity in the results, which indicate, in summary, that cool and dry conditions were potential factors to the spread of COVID-19, and warmer and wetter climates are less likely to enhance the transmissibility of the virus, the certainty of evidence could not be rated lower than that.

The analyses of COVID-19 outbreaks in relation to meteorology aspects reveal significant connection between the incidence of positive cases and climatic conditions. Social factors in combination with meteorological factors play a role in coronavirus outbreaks, since this public health problem is too complex to be explained solely in relation of climatic conditions. Isolation programs, social distancing, number of inhabitants per household, immigration control program, personal hygiene conditions are some of the confounding variables that must interfere in the spread of the new coronavirus, as it occurs with another coronaviruses in the past [38, 39]. Studies that have explored the role of temperature and humidity on the global spread of the disease are expected to take into account public policies, as these measures must be rapidly implemented as an emergency basis of this public health problem, especially to vulnerable countries [40, 41].

In fifteen included studies [9, 20, 22–29, 31–35], the authors investigate the association between temperature and humidity in the transmission rate of COVID-19. In the other two articles [21, 30], the association was made only with temperature. Although humidity has been measured in different ways, like relative and absolute, the great majority of the included studies indicate that there is a relationship between this variable and the spread of the virus.

Luo et al. suggested that sustained transmission and rapid (exponential) growth of cases are possible over a range of humidity and temperature conditions, associated with lack of extensive public health interventions, especially in most social and climatic vulnerable countries [26]. Bu et al. concluded that a temperature range of 13~19°C and humidity of 50% ~ 80% are suitable for the survival and transmission of COVID-19 [22]. Moreover, Wang et al. have support the role that temperature could have in changing the COVID-19 human-to-human transmission and that there might be an optimal temperature for the viral transmission [30]. They suggested that colder regions in the world should adopt the strictest social control measures, since low temperatures significantly contribute to the viability, transmission rate and survival of coronaviruses. Even if the collected data of the study was taken before the outbreak in China, the authors’ results would be extrapolated to the present days and directly interfere the association of temperature and humidity and the public policy in the spread of COVID-19 [22]. Finally, Kathabbi et al. stated that by air quality analysis in areas highly contaminated by the virus, the population could be informed and be encouraged to avoid this area, creating a microclimate that helps to eliminate the spread of COVID-19 [34].

Araújo et al. made it clear that it is not possible to characterize the exact local temperature and humidity conditions that minimizing the virus spread [9]. On the contrary, it is reasonable to determine the type of macroclimate conditions in the places where transmission is occurring. For example, in the tropics, where high temperatures and humidity characterize the weather, the climatic suitability for spread of COVID-19 seems to be more difficult. Heat intolerance of the virus is probably related to the breakdown of their lipid bilayer [42], in a similar model to what occurs with the predecessor of the new coronavirus, the SARS-CoV [43]. Speculative explanation also based on patterns observed for other SARS-CoV justifies the effect of humidity on the less effective spread of the virus on the environment, which reduces the total indirect and secondary transmission [44]. Although higher humidity may increase the atmospheric suspended matter [22], the amount of virus deposited on surfaces, and virus survival time in droplets on surfaces [44], the reduction of the virus spread by indirect air transmission may be an important factor behind the reduced spreading of COVID-19 in a humid climate.

High temperature and high humidity reduce the transmission of others infections of the respiratory tract, like influenza [45, 46] and of SARS coronavirus [11, 39]. The main reasons are: the virus is more stable in cold temperatures, and respiratory droplets, as containers of viruses, remain in suspension longer in dry air [47]. Cold and dry weather can also demote the hosts’ immunity and make them more susceptible to the virus [48].

Many respiratory pathogens show seasonality and the human activity patterns and immunity can be influenced by environmental factors limited during the COVID-19 outbreak, due to the absence of extreme climatic conditions and specific immunity for a newly emerging virus [35]. Cold air temperature contributes to spread of viruses, including coronavirus, and the possibility of infection. Some possible reasons are: low temperature provides suitable survival and reproduction conditions for coronavirus [49]; cold air causes vasoconstriction of the respiratory tract which contributes to weakening of the immune system; and dry cold air makes the nasal mucosa prone to small ruptures, thereby creating opportunities for virus invasion [50]. In contrast, a long period of low or extremely cold temperature played a positive role in reducing the transmission of the coronavirus [9, 22].

In addition, Bukhari et al. discuss another hypothesis for the lower number of COVID-19 cases detected in the tropics [23]. It could be due to less mass testing as many of the underdeveloped countries that presents deficiency in the health care system and may have not done enough testing to detect the actual spread of this virus.

Two of the articles were classified as retrospective and prospective [24, 25], since they suggest implementing future public policies and mass actions that aim to control the spread of COVID-19 around the world. Chen et al. proposed a daily predictive model that in combination with weather observations in the previous 14 days, for five high-latitude cities (New York, Toronto, Italy, Paris and Cologne), is able to predict daily new case counts of COVID-19 for the following 12 days in these places [24]. It is important to notice that a single weather factor alone could not affect the virus transmission too much. However, the combination of different meteorological variables could fit a more complex model, in order to address the systematic influence of different types of weather data on the spread of the virus. Jiwei et al. verified further control with specific medicines and an effective vaccine. Considering the practice of social isolation, the process of this strategy is considered a short-time vaccine for susceptible populations in helping to control the disease [25].

According to Oliveiros et al., temperature and humidity contribute to a maximum of 18% of the variation, the remaining 82% being related to other factors such as containment measures, general health policies, population density, transportation, and cultural aspects [27]. Population migration is another key factor in the spread process that cannot be ignored [25], as well as community structure, social dynamics, and global connectivity [23]. In cities with higher levels of population density, the virus is expected to spread faster than that in less crowded cities [31].

Al-Rousan et al. suggested that international governments should adopt rigorous public policies. This can be done by increasing the number of health professionals and hospitals, and socially isolating, mainly the suspected cases [20]. Better health care facilities tend to reduce the transmission of COVID-19 [31]. The relatively fast outbreak, associated with imperfect daily reporting practices, make a vast underreporting of new cases of COVID-19. Travel limitations and other control interventions need to be implemented consistently [28]. Additionally, Gupta recommended that all citizens be required to wear a face mask whenever they go out, because the primarily viral infection is through airborne or close contact. The Chinese government used this as a key tool in managing the disease, especially with asymptomatic infected people [33].

Finally, the review process of the selected articles should be evaluated with caution. Sixteen of the included studies in this systematic review have not yet gone through the peer review process, which must be taken into consideration when making any inference from the authors’ conclusion [9, 20–24, 26–35].

The results of this systematic review indicate that the confounding variables, together, are even more significant than temperature and relative humidity. The timing, implementation, and magnitude of the likely public interventions from international governments should reduce the adverse consequences of COVID-19 on the public health system. Additionally, it is of great value to the society the knowledge about the lowest rate of transmission of the virus in hot and humid weather and the need of more consistent social isolation in cold and dry weather countries, while there is no treatment protocol established by WHO, as well as the vaccine for COVID-19. The arrival of summer in Europe and in the northern hemisphere are interesting points to consider when loosening slowly the isolation policies. Only with proper planning, unnecessary damage will be avoided for individuals and for the global economy.

### Limitations

The identification of confounding variables and their association with the public health policies were an important limitation of this systematic review. Some studies [9, 20–24, 26, 29, 30, 32, 34, 35] have not evaluated these factors that could influence the impact of climatic variables on the spread of COVID-19, such as migration patterns, containment measures, general health policies, population density, herd immunity, transportation, and cultural aspects. The articles that included these variables in their analysis [25, 27, 28, 31, 33] concluded that climate alone does not explain most of the variability in the spread of the disease. The simple correlation statistical design employed in many articles was not appropriate, which impacted negatively on the classification of risk of bias and certainty of evidence [9, 20, 22, 23, 25, 29, 32]. Furthermore, the selected studies included less than four months of data in their analyzes, not including the main effects in the United States and Europe. However, once again we emphasize the almost unanimity of the conclusions among the included studies, indicating a more modest role of temperature and humidity on the spread of the virus. In addition, several manuscripts did not go through peer review [9, 20–24, 26–35], due to the urgency of publication on the topic, so care should be taken when considering the results of these studies, although they point in the same direction of effect and impact. Finally, since it does not fit the eligibility criteria adopted to conduct this study, the effect of temperature and humidity on the rate of quantitative and qualitative progression of the pandemic could not be estimated, especially when considering the arrival of summer in the countries of Europe and in the northern hemisphere.

## Conclusion

Based on a low level of evidence, the spread of COVID-19 seems to be lower in warm and wet climates. Furthermore, temperature and humidity alone do not explain most of the variability of the COVID-19 outbreak. Public isolation policies, herd immunity, migration patterns, population density, and cultural aspects might directly influence how the spread of this disease occurs. Thus, weather conditions associated with the health policies is a knowledge of great value for the benefit of the humanity in this critical period.

## Supporting information

S1 Checklist PRISMA 2009 checklist. (DOC)

S1 File Search strategy used for each database. (DOCX)
