# Supplementary material for: Effects of temperature and humidity on the spread of COVID-19: A systematic review
Source: PLoS One. 2020 Sep 18;15(9):e0238339. doi: 10.1371/journal.pone.0238339 (PMC7500589; doi:10.1371/journal.pone.0238339)
Supplement: S1 File — (DOCX) [file pone.0238339.s002.docx]

| **Supplementary material 2.** Search strategy for electronic databases. | | |
| --- | --- | --- |
| **Database** | **Search strategy** | **Results** |
| PubMed | (((((((((((((((((((((((((((((((((((((((((((((((((((((((COVID-19[MeSH Terms]) OR COVID-19[Title/Abstract]) OR 2019 novel coronavirus infection[Title/Abstract]) OR COVID19[Title/Abstract]) OR coronavirus disease 2019[Title/Abstract]) OR coronavirus disease-19[Title/Abstract]) OR 2019-nCoV disease[Title/Abstract]) OR 2019 novel coronavirus disease[Title/Abstract]) OR 2019-nCoV infection[Title/Abstract]) OR Coronavirus[MeSH Terms]) OR Coronavirus[Title/Abstract]) OR Coronaviruses[Title/Abstract]) OR Betacoronavirus[MeSH Terms]) OR Betacoronavirus[Title/Abstract]) OR Betacoronaviruses[Title/Abstract]) OR SARS Virus[MeSH Terms]) OR SARS Virus[Title/Abstract]) OR Severe Acute Respiratory Syndrome Virus[Title/Abstract]) OR SARS-Related Coronavirus[Title/Abstract]) OR Coronavirus, SARS-Related[Title/Abstract]) OR SARS Related Coronavirus[Title/Abstract]) OR SARS-CoV[Title/Abstract]) OR Urbani SARS-Associated Coronavirus[Title/Abstract]) OR Coronavirus, Urbani SARS-Associated[Title/Abstract]) OR SARS-Associated Coronavirus, Urbani[Title/Abstract]) OR Urbani SARS Associated Coronavirus[Title/Abstract]) OR SARS Coronavirus[Title/Abstract]) OR Coronavirus, SARS[Title/Abstract]) OR Severe acute respiratory syndrome-related coronavirus[Title/Abstract]) OR Severe acute respiratory syndrome related coronavirus[Title/Abstract]) OR SARS-Associated Coronavirus[Title/Abstract]) OR Coronavirus, SARS-Associated[Title/Abstract]) OR SARS Associated Coronavirus[Title/Abstract]) OR severe acute respiratory syndrome coronavirus 2[MeSH Terms]) OR severe acute respiratory syndrome coronavirus 2[Title/Abstract]) OR Wuhan coronavirus[Title/Abstract]) OR Wuhan seafood market pneumonia virus[Title/Abstract]) OR COVID19 virus[Title/Abstract]) OR COVID-19 virus[Title/Abstract]) OR coronavirus disease 2019 virus[Title/Abstract]) OR SARS-CoV-2[Title/Abstract]) OR SARS2[Title/Abstract]) OR 2019-nCoV[Title/Abstract]) OR 2019 novel coronavirus[Title/Abstract]) OR Coronavirus Infections[MeSH Terms]) OR Coronavirus Infections[Title/Abstract]) OR Coronavirus Infection[Title/Abstract]) OR Infection, Coronavirus[Title/Abstract]) OR Infections, Coronavirus[Title/Abstract]) OR 2019-nCoV[Title/Abstract]) OR New coronavirus[Title/Abstract]) OR 2019-novel-coronavirus[Title/Abstract]) AND ("2019/01/01"[PDat] : "2020/12/31"[PDat]))) AND (((((((((((((((((((((((((((Weather[MeSH Terms]) OR Weather[Title/Abstract]) OR Fog[Title/Abstract]) OR Fogs[Title/Abstract]) OR Climate[MeSH Terms]) OR Climate[Title/Abstract]) OR Climates[Title/Abstract]) OR Humidity[MeSH Terms]) OR Humidity[Title/Abstract]) OR Humidities[Title/Abstract]) OR Temperature[MeSH Terms]) OR Temperature[Title/Abstract]) OR Temperatures[Title/Abstract]) OR Seasons[MeSH Terms]) OR Seasons[Title/Abstract]) OR Season[Title/Abstract]) OR Seasonal Variation[Title/Abstract]) OR Seasonal Variations[Title/Abstract]) OR Variation, Seasonal[Title/Abstract]) OR Variations, Seasonal[Title/Abstract]) OR Altitude[MeSH Terms]) OR Altitude[Title/Abstract]) OR Altitudes[Title/Abstract]) OR Seasonal[Title/Abstract]) OR Seasonality[Title/Abstract]) OR Latitude[Title/Abstract]) AND ("2019/01/01"[PDat] : "2020/12/31"[PDat]))) AND (((((((((((((((((((((((((((((((((((((((((((((((Microbial Viability[MeSH Terms]) OR Microbial Viability[Title/Abstract]) OR Viability, Microbial[Title/Abstract]) OR Virus Viability[Title/Abstract]) OR Viability, Virus[Title/Abstract]) OR Transmission[MeSH Terms]) OR Transmission[Title/Abstract]) OR Infections[MeSH Terms]) OR Infections[Title/Abstract]) OR (Infection[Title/Abstract] AND Infestation[Title/Abstract])) OR (Infestation[Title/Abstract] AND Infection[Title/Abstract])) OR (Infections[Title/Abstract] AND Infestations[Title/Abstract])) OR (Infestations[Title/Abstract] AND Infections[Title/Abstract])) OR Infection[Title/Abstract]) OR Incidence[MeSH Terms]) OR Incidence[Title/Abstract]) OR Incidences[Title/Abstract]) OR Prevalence[MeSH Terms]) OR Prevalence[Title/Abstract]) OR Prevalences[Title/Abstract]) OR Disease Transmission, Infectious[MeSH Terms]) OR Disease Transmission, Infectious[Title/Abstract]) OR Pathogen Transmission[Title/Abstract]) OR Transmission, Pathogen[Title/Abstract]) OR Infectious Disease Transmission[Title/Abstract]) OR Transmission, Infectious Disease[Title/Abstract]) OR Transmission of Infectious Disease[Title/Abstract]) OR Infection Transmission[Title/Abstract]) OR Transmission, Infection[Title/Abstract]) OR Communicable Disease Transmission[Title/Abstract]) OR Disease Transmission, Communicable[Title/Abstract]) OR Transmission, Communicable Disease[Title/Abstract]) OR Autochthonous Transmission[Title/Abstract]) OR Autochthonous Transmissions[Title/Abstract]) OR Transmission, Autochthonous[Title/Abstract]) OR Transmissions, Autochthonous[Title/Abstract]) OR Infectious Disease Transmission, Horizontal[Title/Abstract]) OR Pathogen Transmission, Horizontal[Title/Abstract]) OR Horizontal Transmission of Infectious Disease[Title/Abstract]) OR Horizontal Transmission of Infection[Title/Abstract]) OR Infection Horizontal Transmission[Title/Abstract]) OR Infection Transmission, Horizontal[Title/Abstract]) OR Viability[Title/Abstract]) OR Transmissibility[Title/Abstract]) OR Spread[Title/Abstract]) OR Propagation[Title/Abstract]) OR Spreading[Title/Abstract]) AND (("2019/01/01"[PDat] : "2020/12/31"[PDat])) | 78 |
| Scopus | ( ( ( TITLE-ABS-KEY ( "COVID-19" ) OR TITLE-ABS-KEY ( "2019 novel coronavirus infection" ) OR TITLE-ABS-KEY ( "COVID19" ) OR TITLE-ABS-KEY ( "coronavirus disease 2019" ) OR TITLE-ABS-KEY ( "coronavirus disease-19" ) OR TITLE-ABS-KEY ( "2019-nCoV disease" ) OR TITLE-ABS-KEY ( "2019 novel coronavirus disease" ) OR TITLE-ABS-KEY ( "2019-nCoV infection" ) OR TITLE-ABS-KEY ( coronavirus ) OR TITLE-ABS-KEY ( coronaviruses ) OR TITLE-ABS-KEY ( betacoronavirus ) OR TITLE-ABS-KEY ( betacoronaviruses ) OR TITLE-ABS-KEY ( "SARS Virus" ) OR TITLE-ABS-KEY ( "Severe Acute Respiratory Syndrome Virus" ) OR TITLE-ABS-KEY ( "SARS-Related Coronavirus" ) OR TITLE-ABS-KEY ( "Coronavirus, SARS-Related" ) OR TITLE-ABS-KEY ( "SARS Related Coronavirus" ) OR TITLE-ABS-KEY ( "SARS-CoV" ) OR TITLE-ABS-KEY ( "Urbani SARS-Associated Coronavirus" ) OR TITLE-ABS-KEY ( "Coronavirus, Urbani SARS-Associated" ) OR TITLE-ABS-KEY ( "SARS-Associated Coronavirus, Urbani" ) OR TITLE-ABS-KEY ( "Urbani SARS Associated Coronavirus" ) OR TITLE-ABS-KEY ( "SARS Coronavirus" ) OR TITLE-ABS-KEY ( "Coronavirus, SARS" ) OR TITLE-ABS-KEY ( "Severe acute respiratory syndrome-related coronavirus" ) OR TITLE-ABS-KEY ( "Severe acute respiratory syndrome related coronavirus" ) ) ) AND ( ( TITLE-ABS-KEY ( "SARS-Associated Coronavirus" ) OR TITLE-ABS-KEY ( "Coronavirus, SARS-Associated" ) OR TITLE-ABS-KEY ( "SARS Associated Coronavirus" ) OR TITLE-ABS-KEY ( "severe acute respiratory syndrome coronavirus 2" ) OR TITLE-ABS-KEY ( "Wuhan coronavirus" ) OR TITLE-ABS-KEY ( "Wuhan seafood market pneumonia virus" ) OR TITLE-ABS-KEY ( "COVID19 virus" ) OR TITLE-ABS-KEY ( "COVID-19 virus" ) OR TITLE-ABS-KEY ( "coronavirus disease 2019 virus" ) OR TITLE-ABS-KEY ( "SARS-CoV-2" ) OR TITLE-ABS-KEY ( sars2 ) OR TITLE-ABS-KEY ( "2019-nCoV" ) OR TITLE-ABS-KEY ( "2019 novel coronavirus" ) OR TITLE-ABS-KEY ( "Coronavirus Infections" ) OR TITLE-ABS-KEY ( "Coronavirus Infection" ) OR TITLE-ABS-KEY ( "Infection, Coronavirus" ) OR TITLE-ABS-KEY ( "Infections, Coronavirus" ) OR TITLE-ABS-KEY ( "2019-nCoV" ) OR TITLE-ABS-KEY ( "New coronavirus" ) OR TITLE-ABS-KEY ( "2019-novel-coronavirus" ) ) ) ) AND ( ( TITLE-ABS-KEY ( weather ) OR TITLE-ABS-KEY ( fog ) OR TITLE-ABS-KEY ( fogs ) OR TITLE-ABS-KEY ( climate ) OR TITLE-ABS-KEY ( climates ) OR TITLE-ABS-KEY ( humidity ) OR TITLE-ABS-KEY ( humidities ) OR TITLE-ABS-KEY ( temperature ) OR TITLE-ABS-KEY ( temperatures ) OR TITLE-ABS-KEY ( seasons ) OR TITLE-ABS-KEY ( season ) OR TITLE-ABS-KEY ( "Seasonal Variation" ) OR TITLE-ABS-KEY ( "Seasonal Variations" ) OR TITLE-ABS-KEY ( "Variation, Seasonal" ) OR TITLE-ABS-KEY ( "Variations, Seasonal" ) OR TITLE-ABS-KEY ( altitude ) OR TITLE-ABS-KEY ( altitudes ) OR TITLE-ABS-KEY ( seasonal ) OR TITLE-ABS-KEY ( seasonality ) OR TITLE-ABS-KEY ( latitude ) ) ) AND ( ( TITLE-ABS-KEY ( "Microbial Viability" ) OR TITLE-ABS-KEY ( "Viability, Microbial" ) OR TITLE-ABS-KEY ( "Virus Viability" ) OR TITLE-ABS-KEY ( "Viability, Virus" ) OR TITLE-ABS-KEY ( transmission ) OR TITLE-ABS-KEY ( infections ) OR TITLE-ABS-KEY ( "Infection and Infestation" ) OR TITLE-ABS-KEY ( "Infestation and Infection" ) OR TITLE-ABS-KEY ( "Infections and Infestations" ) OR TITLE-ABS-KEY ( "Infestations and Infections" ) OR TITLE-ABS-KEY ( infection ) OR TITLE-ABS-KEY ( incidence ) OR TITLE-ABS-KEY ( incidences ) OR TITLE-ABS-KEY ( prevalence ) OR TITLE-ABS-KEY ( prevalences ) OR TITLE-ABS-KEY ( "Disease Transmission, Infectious" ) OR TITLE-ABS-KEY ( "Pathogen Transmission" ) OR TITLE-ABS-KEY ( "Pathogen Transmission" ) OR TITLE-ABS-KEY ( "Infectious Disease Transmission" ) OR TITLE-ABS-KEY ( "Transmission, Infectious Disease" ) OR TITLE-ABS-KEY ( "Transmission of Infectious Disease" ) OR TITLE-ABS-KEY ( "Infection Transmission" ) OR TITLE-ABS-KEY ( "Transmission, Infection" ) OR TITLE-ABS-KEY ( "Communicable Disease Transmission" ) OR TITLE-ABS-KEY ( "Disease Transmission, Communicable" ) OR TITLE-ABS-KEY ( "Transmission, Communicable Disease" ) OR TITLE-ABS-KEY ( "Autochthonous Transmission" ) OR TITLE-ABS-KEY ( "Autochthonous Transmissions" ) OR TITLE-ABS-KEY ( "Transmission, Autochthonous" ) OR TITLE-ABS-KEY ( "Transmissions, Autochthonous" ) OR TITLE-ABS-KEY ( "Infectious Disease Transmission, Horizontal" ) OR TITLE-ABS-KEY ( "Pathogen Transmission, Horizontal" ) OR TITLE-ABS-KEY ( "Horizontal Transmission of Infectious Disease" ) OR TITLE-ABS-KEY ( "Horizontal Transmission of Infection" ) OR TITLE-ABS-KEY ( "Infection Horizontal Transmission" ) OR TITLE-ABS-KEY ( "Infection Transmission, Horizontal" ) OR TITLE-ABS-KEY ( viability ) OR TITLE-ABS-KEY ( transmissibility ) OR TITLE-ABS-KEY ( spread ) OR TITLE-ABS-KEY ( propagation ) OR TITLE-ABS-KEY ( spreading ) ) ) AND ( LIMIT-TO ( PUBYEAR , 2020 ) OR LIMIT-TO ( PUBYEAR , 2019 ) ) | 37 |
| Web of Science | TÓPICO: ("COVID-19") *OR* TÓPICO: ("2019 novel coronavirus infection") *OR* TÓPICO: (COVID19) *OR* TÓPICO: ("coronavirus disease 2019") *OR* TÓPICO: ("coronavirus disease-19") *OR* TÓPICO: ("2019-nCoV disease") *OR* TÓPICO: ("2019 novel coronavirus disease") *OR* TÓPICO: ("2019-nCoV infection") *OR* TÓPICO: (Coronavirus) *OR* TÓPICO: (Coronaviruses) *OR* TÓPICO: (Betacoronavirus) *OR* TÓPICO: (Betacoronaviruses) *OR* TÓPICO: ("SARS Virus") *OR* TÓPICO: ("Severe Acute Respiratory Syndrome Virus") *OR* TÓPICO: ("SARS-Related Coronavirus") *OR* TÓPICO: ("Coronavirus, SARS-Related") *OR* TÓPICO: ("SARS Related Coronavirus") *OR* TÓPICO: ("SARS-CoV") *OR* TÓPICO: ("Urbani SARS-Associated Coronavirus") *OR* TÓPICO: ("Coronavirus, Urbani SARS-Associated") *OR* TÓPICO: ("SARS-Associated Coronavirus, Urbani") *OR* TÓPICO: ("Urbani SARS Associated Coronavirus") *OR* TÓPICO: ("SARS Coronavirus") *OR* TÓPICO: ("Coronavirus, SARS") *OR* TÓPICO: ("Severe acute respiratory syndrome-related coronavirus") *OR* TÓPICO: ("Severe acute respiratory syndrome related coronavirus") *OR* TÓPICO: ("SARS-Associated Coronavirus") *OR* TÓPICO: ("Coronavirus, SARS-Associated") *OR* TÓPICO: ("SARS Associated Coronavirus") *OR* TÓPICO: ("severe acute respiratory syndrome coronavirus 2") *OR* TÓPICO: ("Wuhan coronavirus") *OR* TÓPICO: ("Wuhan seafood market pneumonia virus") *OR* TÓPICO: ("COVID19 virus") *OR* TÓPICO: ("COVID-19 virus") *OR* TÓPICO: ("coronavirus disease 2019 virus") *OR* TÓPICO: ("SARS-CoV-2") *OR* TÓPICO: (SARS2) *OR* TÓPICO: ("2019-nCoV") *OR* TÓPICO: ("2019 novel coronavirus") *OR* TÓPICO: ("Coronavirus Infections") *OR* TÓPICO: ("Coronavirus Infection") *OR* TÓPICO: ("Infection, Coronavirus") *OR* TÓPICO: ("Infections, Coronavirus") *OR* TÓPICO: ("2019-nCoV") *OR* TÓPICO: ("New coronavirus") *OR* TÓPICO: ("2019-novel-coronavirus") *AND* TÓPICO: (Weather) *OR* TÓPICO: (Fog) *OR* TÓPICO: (Fogs) *OR* TÓPICO: (Climate) *OR* TÓPICO: (Climates) *OR* TÓPICO: (Humidity) *OR* TÓPICO: (Humidities) *OR* TÓPICO: (Temperature) *OR* TÓPICO: (Temperatures) *OR* TÓPICO: (Seasons) *OR* TÓPICO: (Season) *OR* TÓPICO: ("Seasonal Variation") *OR* TÓPICO: ("Seasonal Variations") *OR* TÓPICO: ("Variation, Seasonal") *OR* TÓPICO: ("Variations, Seasonal") *OR* TÓPICO: (Altitude) *OR* TÓPICO: (Altitudes) *OR* TÓPICO: (Seasonal) *OR* TÓPICO: (Seasonality) *OR* TÓPICO: (Latitude) *AND* TÓPICO: ("Microbial Viability") *OR* TÓPICO: ("Viability, Microbial") *OR* TÓPICO: ("Virus Viability") *OR* TÓPICO: ("Viability, Virus") *OR* TÓPICO: (Transmission) *OR* TÓPICO: (Infections) *OR* TÓPICO: ("Infection and Infestation") *OR* TÓPICO: ("Infestation and Infection") *OR* TÓPICO: ("Infections and Infestations") *OR* TÓPICO: ("Infestations and Infections") *OR* TÓPICO: (Infection) *OR* TÓPICO: (Incidence) *OR* TÓPICO: (Incidences) *OR* TÓPICO: (Prevalence) *OR* TÓPICO: (Prevalences) *OR* TÓPICO: ("Disease Transmission, Infectious") *OR* TÓPICO: ("Pathogen Transmission") *OR* TÓPICO: ("Transmission, Pathogen") *OR* TÓPICO: ("Infectious Disease Transmission") *OR* TÓPICO: ("Transmission, Infectious Disease") *OR* TÓPICO: ("Transmission of Infectious Disease") *OR* TÓPICO: ("Infection Transmission") *OR* TÓPICO: ("Transmission, Infection") *OR* TÓPICO: ("Communicable Disease Transmission") *OR* TÓPICO: ("Disease Transmission, Communicable") *OR* TÓPICO: ("Transmission, Communicable Disease") *OR* TÓPICO: ("Autochthonous Transmission") *OR* TÓPICO: ("Autochthonous Transmissions") *OR* TÓPICO: ("Transmission, Autochthonous") *OR* TÓPICO: ("Transmissions, Autochthonous") *OR* TÓPICO: ("Infectious Disease Transmission, Horizontal") *OR* TÓPICO: ("Pathogen Transmission, Horizontal") *OR* TÓPICO: ("Horizontal Transmission of Infectious Disease") *OR* TÓPICO: ("Horizontal Transmission of Infection") *OR* TÓPICO: ("Infection Horizontal Transmission") *OR* TÓPICO: ("Infection Transmission, Horizontal") *OR* TÓPICO: (Viability) *OR* TÓPICO: (Transmissibility) *OR* TÓPICO: (Spread) *OR* TÓPICO: (Propagation Spreading) *OR* TÓPICO: (Spreading) | 71 |
| Cochrane | #1 ("COVID-19"):ti,ab,kw OR ("2019 novel coronavirus infection"):ti,ab,kw OR (COVID19):ti,ab,kw OR ("coronavirus disease 2019"):ti,ab,kw OR ("coronavirus disease-19"):ti,ab,kw (Word variations have been searched)  #2 ("2019-nCoV disease"):ti,ab,kw OR ("2019 novel coronavirus disease"):ti,ab,kw OR ("2019-nCoV infection"):ti,ab,kw OR (Coronavirus):ti,ab,kw OR (Coronaviruses):ti,ab,kw (Word variations have been searched)  #3 (Betacoronavirus):ti,ab,kw OR (Betacoronaviruses):ti,ab,kw OR ("SARS Virus"):ti,ab,kw OR ("Severe Acute Respiratory Syndrome Virus"):ti,ab,kw OR ("SARS-Related Coronavirus"):ti,ab,kw (Word variations have been searched)  #4 ("Coronavirus, SARS-Related"):ti,ab,kw OR ("SARS Related Coronavirus"):ti,ab,kw OR ("SARS-CoV"):ti,ab,kw OR ("Urbani SARS-Associated Coronavirus"):ti,ab,kw OR ("Coronavirus, Urbani SARS-Associated"):ti,ab,kw (Word variations have been searched)  #5 ("SARS-Associated Coronavirus, Urbani"):ti,ab,kw OR ("Urbani SARS Associated Coronavirus"):ti,ab,kw OR ("SARS Coronavirus"):ti,ab,kw OR ("Coronavirus, SARS"):ti,ab,kw OR ("Severe acute respiratory syndrome-related coronavirus"):ti,ab,kw (Word variations have been searched)  #6 ("Severe acute respiratory syndrome related coronavirus"):ti,ab,kw OR ("SARS-Associated Coronavirus"):ti,ab,kw OR ("Coronavirus, SARS-Associated"):ti,ab,kw OR ("SARS Associated Coronavirus"):ti,ab,kw OR ("severe acute respiratory syndrome coronavirus 2"):ti,ab,kw (Word variations have been searched)  #7 ("Wuhan coronavirus"):ti,ab,kw OR ("Wuhan seafood market pneumonia virus"):ti,ab,kw OR ("COVID19 virus"):ti,ab,kw OR ("COVID-19 virus"):ti,ab,kw OR ("coronavirus disease 2019 virus"):ti,ab,kw (Word variations have been searched)  #8 ("SARS-CoV-2"):ti,ab,kw OR (SARS2):ti,ab,kw OR ("2019-nCoV"):ti,ab,kw OR ("2019 novel coronavirus"):ti,ab,kw OR ("Coronavirus Infections"):ti,ab,kw (Word variations have been searched)  #9 ("Coronavirus Infection"):ti,ab,kw OR ("Infection, Coronavirus"):ti,ab,kw OR ("Infections, Coronavirus"):ti,ab,kw OR ("2019-nCoV"):ti,ab,kw (Word variations have been searched)  #10 ("New coronavirus"):ti,ab,kw OR ("2019-novel-coronavirus"):ti,ab,kw (Word variations have been searched)  #11 #1 OR #2 OR #3 OR #4 OR #5 OR #6 OR #7 OR #8 OR #9 OR #10  #12 (Weather):ti,ab,kw OR (Fog):ti,ab,kw OR (Fogs):ti,ab,kw OR (Climate):ti,ab,kw OR (Climates):ti,ab,kw (Word variations have been searched)  #13 (Humidity):ti,ab,kw OR (Humidities):ti,ab,kw OR (Temperature):ti,ab,kw OR (Temperatures):ti,ab,kw OR (Seasons):ti,ab,kw (Word variations have been searched)  #14 (Season):ti,ab,kw OR ("Seasonal Variation"):ti,ab,kw OR ("Seasonal Variations"):ti,ab,kw OR ("Variation, Seasonal"):ti,ab,kw OR ("Variations, Seasonal"):ti,ab,kw (Word variations have been searched)  #15 (Altitude):ti,ab,kw OR (Altitudes):ti,ab,kw OR (Seasonal):ti,ab,kw OR (Seasonality):ti,ab,kw OR (Latitude):ti,ab,kw (Word variations have been searched)  #16 #12 OR #13 OR #14 OR #15  #17 ("Microbial Viability"):ti,ab,kw OR ("Viability, Microbial"):ti,ab,kw OR ("Virus Viability"):ti,ab,kw OR ("Viability, Virus"):ti,ab,kw OR (Transmission):ti,ab,kw (Word variations have been searched)  #18 (Infections):ti,ab,kw OR ("Infection and Infestation"):ti,ab,kw OR ("Infestation and Infection"):ti,ab,kw OR ("Infections and Infestations"):ti,ab,kw OR ("Infestations and Infections"):ti,ab,kw (Word variations have been searched)  #19 (Infection):ti,ab,kw OR (Incidence):ti,ab,kw OR (Incidences):ti,ab,kw OR (Prevalence):ti,ab,kw OR (Prevalences):ti,ab,kw (Word variations have been searched)  #20 ("Disease Transmission, Infectious"):ti,ab,kw OR ("Pathogen Transmission"):ti,ab,kw OR ("Transmission, Pathogen"):ti,ab,kw OR ("Infectious Disease Transmission"):ti,ab,kw OR ("Transmission, Infectious Disease"):ti,ab,kw (Word variations have been searched)  #21 ("Transmission of Infectious Disease"):ti,ab,kw OR ("Infection Transmission"):ti,ab,kw OR ("Transmission, Infection"):ti,ab,kw OR ("Communicable Disease Transmission"):ti,ab,kw OR ("Disease Transmission, Communicable"):ti,ab,kw (Word variations have been searched)  #22 ("Transmission, Communicable Disease"):ti,ab,kw OR ("Autochthonous Transmission"):ti,ab,kw OR ("Autochthonous Transmissions"):ti,ab,kw OR ("Transmission, Autochthonous"):ti,ab,kw OR ("Transmissions, Autochthonous"):ti,ab,kw (Word variations have been searched)  #23 ("Infectious Disease Transmission, Horizontal"):ti,ab,kw OR ("Pathogen Transmission, Horizontal"):ti,ab,kw OR ("Horizontal Transmission of Infectious Disease"):ti,ab,kw OR ("Horizontal Transmission of Infection"):ti,ab,kw OR ("Infection Horizontal Transmission"):ti,ab,kw (Word variations have been searched)  #24 ("Infection Transmission, Horizontal"):ti,ab,kw OR (Viability):ti,ab,kw OR (Transmissibility):ti,ab,kw (Word variations have been searched)  #25 (Spread):ti,ab,kw OR (Propagation):ti,ab,kw OR (Spreading):ti,ab,kw (Word variations have been searched)  #26 #17 OR #18 OR #19 OR #20 OR #21 OR #22 OR #23 OR #24 OR #25  #27 #11 AND #16 AND #26 | 2 |
| LILACS | (tw:((tw:(COVID-19)) OR (tw:(2019 novel coronavirus infection)) OR (tw:(COVID19)) OR (tw:(coronavirus disease 2019)) OR (tw:(coronavirus disease-19)) OR (tw:(2019-nCoV disease)) OR (tw:(2019 novel coronavirus disease)) OR (tw:(2019-nCoV infection)) OR (tw:(Coronavirus)) OR (tw:(Coronaviruses)) OR (tw:(Betacoronavirus)) OR (tw:(Betacoronaviruses)) OR (tw:(SARS Virus)) OR (tw:(Severe Acute Respiratory Syndrome Virus)) OR (tw:(SARS-Related Coronavirus)) OR (tw:(Coronavirus, SARS-Related)) OR (tw:(SARS Related Coronavirus)) OR (tw:(SARS-CoV)) OR (tw:(Urbani SARS-Associated Coronavirus)) OR (tw:(Coronavirus, Urbani SARS-Associated)) OR (tw:(SARS-Associated Coronavirus, Urbani)) OR (tw:(Urbani SARS Associated Coronavirus)) OR (tw:(SARS Coronavirus)) OR (tw:(Coronavirus, SARS)) OR (tw:(Severe acute respiratory syndrome-related coronavirus)) OR (tw:(Severe acute respiratory syndrome related coronavirus)) OR (tw:(SARS-Associated Coronavirus)) OR (tw:(Coronavirus, SARS-Associated)) OR (tw:(SARS Associated Coronavirus)) OR (tw:(severe acute respiratory syndrome coronavirus 2)) OR (tw:(Wuhan coronavirus)) OR (tw:(Wuhan seafood market pneumonia virus)) OR (tw:(COVID19 virus)) OR (tw:(COVID-19 virus)) OR (tw:(coronavirus disease 2019 virus)) OR (tw:(SARS-CoV-2)) OR (tw:(SARS2)) OR (tw:(2019-nCoV)) OR (tw:(2019 novel coronavirus)) OR (tw:(Coronavirus Infections)) OR (tw:(Coronavirus Infection)) OR (tw:(Infection, Coronavirus)) OR (tw:(Infections, Coronavirus)) OR (tw:(2019-nCoV)) OR (tw:(New coronavirus)) OR (tw:(2019-novel-coronavirus)))) AND (tw:((tw:(Weather)) OR (tw:(Fog)) OR (tw:(Fogs)) OR (tw:(Climate)) OR (tw:(Climates)) OR (tw:(Humidity)) OR (tw:(Humidities)) OR (tw:(Temperature)) OR (tw:(Temperatures)) OR (tw:(Seasons)) OR (tw:(Season)) OR (tw:(Seasonal Variation)) OR (tw:(Seasonal Variations)) OR (tw:(Variation, Seasonal)) OR (tw:(Variations, Seasonal)) OR (tw:(Altitude)) OR (tw:(Altitudes)) OR (tw:(Seasonal)) OR (tw:(Seasonality)) OR (tw:(Latitude)))) AND (tw:((tw:(Microbial Viability)) OR (tw:(Viability, Microbial)) OR (tw:(Virus Viability)) OR (tw:(Viability, Virus)) OR (tw:(Transmission)) OR (tw:(Infections)) OR (tw:(Infection and Infestation)) OR (tw:(Infestation and Infection)) OR (tw:(Infections and Infestations)) OR (tw:(Infestations and Infections)) OR (tw:(Infection)) OR (tw:(Incidence)) OR (tw:(Incidences)) OR (tw:(Prevalence)) OR (tw:(Prevalences)) OR (tw:(Disease Transmission, Infectious)) OR (tw:(Pathogen Transmission)) OR (tw:(Transmission, Pathogen)) OR (tw:(Infectious Disease Transmission)) OR (tw:(Transmission, Infectious Disease)) OR (tw:(Transmission of Infectious Disease)) OR (tw:(Infection Transmission)) OR (tw:(Transmission, Infection)) OR (tw:(Communicable Disease Transmission)) OR (tw:(Disease Transmission, Communicable)) OR (tw:(Transmission, Communicable Disease)) OR (tw:(Autochthonous Transmission)) OR (tw:(Autochthonous Transmissions)) OR (tw:(Transmission, Autochthonous)) OR (tw:(Transmissions, Autochthonous)) OR (tw:(Infectious Disease Transmission, Horizontal)) OR (tw:(Pathogen Transmission, Horizontal)) OR (tw:(Horizontal Transmission of Infectious Disease)) OR (tw:(Horizontal Transmission of Infection)) OR (tw:(Infection Horizontal Transmission)) OR (tw:(Infection Transmission, Horizontal)) OR (tw:(Viability)) OR (tw:(Transmissibility)) OR (tw:(Spread)) OR (tw:(Propagation)) OR (tw:(Spreading)))) | 4 |
| OpenGrey | coronavirus AND spread | 0 |
| Google Scholar | coronavirus AND weather AND spread | 325 |
